# Revisiting a Chinese Endemic Termite Genus of Building Timber Pests: Mitochondrial Genomic and Morphological Dissection and Phylogenetic Positioning of *Xiaitermes* Gao & He, 1994 (Termitidae: Nasutitermitinae)

**DOI:** 10.3390/insects17060602

**Published:** 2026-06-08

**Authors:** Fei Ye, Yun-Ling Ke, Zhi-Qiang Li

**Affiliations:** Guangdong Key Laboratory of Animal Conservation and Resource Utilization, Institute of Zoology, Guangdong Academy of Sciences, Guangzhou 510260, China; yefei@giz.gd.cn (F.Y.); keyl@giz.gd.cn (Y.-L.K.)

**Keywords:** Isoptera, *Xiaitermes*, morphometric trait, mitochondrial genome, mitophylogenomics

## Abstract

*Xiaitermes* Gao & He, 1994 is a small genus within Nasutitermitinae, the largest subfamily of Termitidae. It comprises only two species, both of which are wood-feeding pests endemic to China that damage building timbers and economic crops. However, the morphological similarity between these two species, combined with a lack of molecular data, has made species identification challenging. This study determined the first mitochondrial genomes of *Xiaitermes tiantaiensis* Gao & He, 1994 and *Xiaitermes yinxianensis* Gao & He, 1994 (15,867 bp and 15,866 bp in length, respectively), which share 99.9% sequence identity. Comparative mitogenomic analysis revealed an A+T-biased nucleotide composition and codon usage pattern, as well as conserved tRNA secondary structures. Morphological analysis of the soldier caste demonstrated that the apical processes of the mandibles exhibit considerable intraspecific variation and are not diagnostic for species delimitation. Furthermore, the ranges of the seven morphological measurements strongly overlapped. Integrated molecular and morphological evidence confirmed that *X. tiantaiensis* is a junior synonym of *X. yinxianensis*. Although phylogenetic analyses based on mitogenomic data only partially resolved relationships among subfamilies within Termitidae, they provided better resolution within Nasutitermitinae and consistently placed *Xiaitermes* as closely related to *Ahmaditermes*.

## Simple Summary

Termitidae constitute the largest lineage of termites, functioning as key decomposers in terrestrial ecosystems while also encompassing certain pest species. *Xiaitermes* Gao & He, 1994, a Chinese endemic genus in the most diverse subfamily Nasutitermitinae, comprises only two wood-feeding species that damage building timbers and economic crops. However, morphological identification of these species remains challenging, and no molecular data are available to date, impeding accurate diagnosis in pest management. This study reports the first *Xiaitermes* mitochondrial genomes and comprehensive comparative analyses of molecular and morphological data, revealing the synonymy of the two species and their phylogenetic position within Nasutitermitinae. These findings not only facilitate rapid pest identification but also improve our understanding of the phylogeny of Nasutitermitinae.

## 1. Introduction

Termites are a relatively small but ecologically and economically significant insect clade, with approximately 280 genera and 3000 described species assigned to 13 families [1,2]. Owing to their considerable biomass and ability to digest lignocellulose [3], termites, particularly as dominant soil invertebrates in the tropics and subtropics, exert a profound influence on habitat soil formation, structural and chemical properties, and global biogeochemical cycles [4,5,6], resulting in their designation as soil ecosystem engineers. Moreover, approximately 12% of termite species are significant pests, some of which are invasive worldwide, causing severe damage to building timber, water conservancy facilities, and agricultural crops [7,8,9].

As the most diverse and highly evolved group, Termitidae constitute ~80% of termite species (over 2000 species in 18 subfamilies [2]), and consequently harbor the most pest species. *Xiaitermes* Gao & He, 1994 is a small, endemic Chinese genus in the subfamily Nasutitermitinae, comprising two xylophagous species (*Xiaitermes yinxianensis* Gao & He, 1994 and *Xiaitermes tiantaiensis* Gao & He, 1994), both of which are building timber pests [10]. In addition, *X. yinxianensis* also inflicts damage on agricultural crops such as Chinese bayberry *Myrica rubra* [11]. Both species are primarily and sympatrically distributed in Zhejiang Province, eastern China, with *X. yinxianensis* also recorded in the adjacent Anhui Province [12]. The soldiers of *X. yinxianensis* are dimorphic, whereas those of *X. tiantaiensis* are monomorphic. Beyond soldier polymorphism, the original morphological description distinguishes the two as follows: soldiers of *X. yinxianensis* are larger in body size and possess distinct apical processes of mandibles, whereas those of *X. tiantaiensis* are smaller and lack (or have obscure) apical processes of mandibles. However, the apical processes of the soldiers have been demonstrated to be inconsistent taxonomic characteristics across and within several genera in Nasutitermitinae [13]. The potential instability of key diagnostic characteristics and their highly sympatric distributions make accurate pest identification challenging and indicate that the relationship between both species requires further validation with multiple lines of evidence. Nevertheless, for more than three decades since its description, apart from pest or biodiversity surveys, this pest genus has never been subjected to any morphological or systematic investigations.

Termite morphological identification has long represented a significant challenge [1,14], particularly for nonexperts, while the adoption of molecular methods, such as DNA barcoding, has substantially improved the accuracy and efficiency of species identification. Although several gene fragments, such as *COII* and *rrnL*, have served as universal DNA barcode markers for termites [15,16] with broadest taxonomic coverage, the mitochondrial genome (mitogenome), with the key advantage of providing significantly better resolution and precision than traditional markers for species delimitation and phylogenetic reconstruction, has become the marker of choice in such research over the past decade [17,18,19]. However, the complete dearth of molecular data for many endemic lineages, including *Xiaitermes*, compounded by their uncertain phylogenetic placement within Nasutitermitinae, reflects both the persistent data imbalance regarding endemic pests and the correspondingly unresolved phylogeny, highlighting the urgent need to increase genetic sampling for such neglected lineages.

In this study, we determined the first *Xiaitermes* mitogenomes and conducted a comprehensive comparative analysis integrating morphological and genomic data to clarify their relationships. Furthermore, we reconstructed the phylogeny of Termitidae on the basis of mitogenomes, focusing specifically on inferring the phylogenetic placement of *Xiaitermes*.

## 2. Materials and Methods

### 2.1. Specimen Collection and Morphological Examinations

One colony each of *X. tiantaiensis* and *X. yinxianensis* was collected from Zhejiang Province (121°19′28″ E, 29°22′49″ N, Ninghai, Ningbo, July 2014) and Anhui Province (117°31′41″ E, 29°36′56″ N, Qimen, Huangshan, 9 June 2023). These samples were preserved in absolute ethanol and stored at −40 °C until morphological observation and DNA extraction. Type specimens of *X. tiantaiensis* (three soldiers) and *X. yinxianensis* (three major soldiers) received from Shanghai Entomological Museum, Chinese Academy of Sciences were preserved in 75% ethanol for morphological analysis. All these type specimens and voucher specimens were deposited at the Institute of Zoology, Guangdong Academy of Sciences, Guangzhou, China.

Specimens were observed, measured, and identified using a stereomicroscope (SAPO, Leica, Wetzlar, Germany) based on keys provided by Huang et al. [20]. Images of the soldiers were recorded using a VHX-X1 digital microscope (Keyence, Osaka, Japan). A total of seven quantitative characteristics of the soldier caste, encompassing all traits mentioned in the original description by He and Gao [10]—including head length to base of mandibles, head length with rostrum, maximum head width, head height with postmentum, maximum pronotum length, maximum pronotum width, and hind tibia length—were measured for *X. tiantaiensis* (soldier: *n* = 10 including type specimens) and *X. yinxianensis* (minor soldier: *n* = 14, major soldier: *n* = 13 including type specimens). To compare the species differences between soldiers of both species, we performed separate non-parametric Kruskal–Wallis tests for each morphological characteristic, followed by Dunn’s test for pairwise comparisons, and conducted principal component analysis (PCA) on the basis of all seven quantitative characteristics.

### 2.2. Mitogenome Generation, Annotation and Analyses

Genomic DNA was extracted from the head and thorax of single worker specimens using the cetyltrimethylammonium bromide method [21]. For each species, short-insert (350 bp) libraries were constructed and sequenced with 150 bp paired-end reads on the DNBSEQ platform at BGI (Shenzhen, China). Following quality control with fastp 0.24.3 [22], 3.60 Gb (*X. tiantaiensis*) and 3.62 Gb (*X. yinxianensis*) of clean data were used for *de novo* assembly using NOVOPlasty 4.3.1 [23] with the *COII* gene, which was amplified with the universal primers A-tLeu and B-tLys [24] and sequenced by the Sanger method, as the seed sequence.

Gene annotation and the secondary structure of transfer RNAs (tRNAs) were initially predicted using MITOS 2 [25]. The boundaries of protein-coding genes (PCGs) and ribosomal RNA (rRNA) genes were subsequently manually corrected by alignment with their homologues in published Nasutitermitinae mitogenomes using MUSCLE 5 [26]. MEGA 11 [27] was used to determine base composition and codon usage, and the formulas AT-skew = (A − T)/(A + T) and GC−skew = (G − C)/(G + C) [28] were applied to assess base compositional asymmetry. The mitogenome map was visualized using Proksee (https://proksee.ca, accessed on 15 March 2026) [29], and other plots were generated using OriginPro 2025 (OriginLab, Northampton, MA, USA). Newly sequenced mitogenomes were deposited into GenBank under the accession numbers PZ210214 and PZ210215.

### 2.3. Phylogenetic Analyses

Taxon sampling included 76 ingroup species of Termitidae, with 48 terminals representing Nasutitermitinae, and three outgroup species of Heterotermitidae (Table S1). Phylogenetic analyses were conducted using maximum likelihood (ML) and Bayesian inference (BI) methods based on two matrices, namely, PCGAARNA and PCGNTRNA. The former matrix was concatenated from the aligned amino acid sequences of the 13 PCGs and the nucleotide sequences of all tRNAs and rRNAs, whereas the latter matrix was composed of the aligned nucleotide sequences of the 37 genes. The PCGs were aligned at the amino acid level using MUSCLE implemented in MEGA and subsequently back-translated to nucleotides, followed by nucleotide alignment of RNA genes with manual refinement guided by secondary structure models [30]. The third codon positions of PCGs were retained because no evidence of saturation was detected using Xia’s method in DAMBE 7 (NumOTU = 32, Iss = 0.421, Iss.cAsym = 0.554) [31,32]. Before concatenation, individual alignments of amino acid and nucleotide sequences were filtered using Gblocks 0.91b [33] to exclude ambiguously aligned sites. The heterogeneity of sequence divergence was assessed using AliGROOVE 1.05 [34] with the default sliding window size, encompassing the amino acid and nucleotide sequences of PCGs along with the nucleotide sequences of tRNA and rRNA genes. Indels in the nucleotide alignments were treated as a fifth state, and a BLOSUM62 matrix was applied for the amino acid substitution matrix.

The best-fit substitution model and partitioning scheme (gene/gene + codon positions of PCGs) were determined using ModelFinder [35], implemented in IQ-TREE 2 [36]. ML analyses were also performed in IQ-TREE, with node support assessed from 2000 ultrafast bootstrap replicates. BI analyses were conducted using MrBayes 2.3.7 [37], which involved performing two independent runs of 20 and 40 million generations for PCGNTRNA and PCGAARNA matrices respectively, sampling every 1000 generations and discarding the first 25% of the samples as burn-in. Convergence was confirmed by the values of an average standard deviation of split frequencies < 0.01.

## 3. Results and Discussion

### 3.1. Mitogenome Architecture and General Characteristics

**Figure 1:**
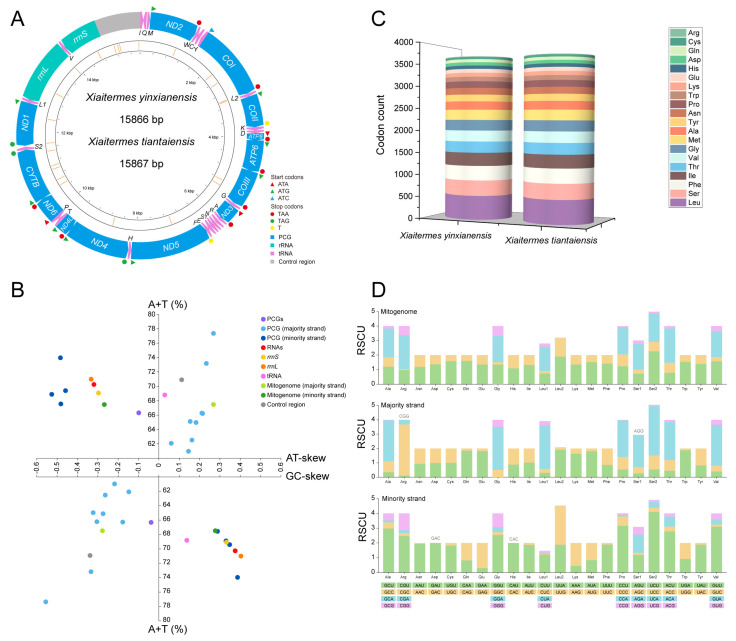
General mitogenomic features of *Xiaitermes*. (**A**) Mitogenomic organization of *Xiaitermes tiantaiensis* and *Xiaitermes yinxianensis*. Variable sites between two species are indicated on the inner track in yellow. (**B**) Nucleotide composition of *Xiaitermes* mitogenomes. (**C**) Codon usage abundance in the mitogenomes of *X. tiantaiensis* and *X. yinxianensis*. (**D**) Relative synonymous codon usage (RSCU) for all PCGs, for PCGs on the majority strand, and for PCGs on the minority strand of the *Xiaitermes* mitogenomes. Absent codons are shown in gray.

The complete mitogenomes of *X. tiantaiensis* and *X. yinxianensis* were circular, double-stranded DNA, with sizes of 15,867 bp and 15,866 bp, respectively. Both mitogenomes contained the conserved metazoan complement of 37 genes, including 13 PCGs, two rRNA genes and 22 tRNA genes (Figure 1A). Fourteen genes were encoded on the minority strand, including eight tRNA genes, two rRNA genes and four PCGs, and the remaining genes were on the majority strand in both species, with an identical gene order—the putative ancestral insect gene order, which is conserved across termites [30,38,39,40]. The *X. tiantaiensis* and *X. yinxianensis* mitogenomes exhibited the typical A+T bias of insect mitogenomes, with A+T contents of 67.47% and 67.49%, respectively. Among the coding regions, the RNA genes presented a higher A+T content than the PCGs did, with the rRNA genes displaying the highest values, a level matching that of the control region (Figure 1B). Furthermore, *Xiaitermes* mitogenomes showed evident A and C bias on the majority strand, attributed to asymmetric mutational constraints during replication [41]. With the exception of the short tRNA genes, all the PCGs and rRNA genes shared a strand-specific base composition bias of their coding strand, with no reversed bias as observed in other insects [42].

The biased nucleotide composition (AT-rich) was mirrored in codon usage patterns in both *Xiaitermes* mitogenomes. The most frequently used codons were A/T-rich (UUU, AUU, AUA, and UUA), whereas fewer G/C-rich codons were used (Figure 1C), which is a pattern conserved in most insect lineages [43]. Furthermore, a strand-specific bias was observed: synonymous codons ending with A/C were favored in majority-strand PCGs, whereas the opposite preference was found in minority-strand PCGs (Figure 1D). *Xiaitermes* mitogenomes also shared identical start and stop codons for each PCG. Additionally, they possessed consistent canonical cloverleaf secondary structures for tRNAs (Figure S1), except for *trnS1*, which lacks the DHU arm, a common feature in metazoan mitochondrial genomes [44].

### 3.2. Phylogeny of Termitidae and the Placement of Xiaitermes

**Figure 2:**
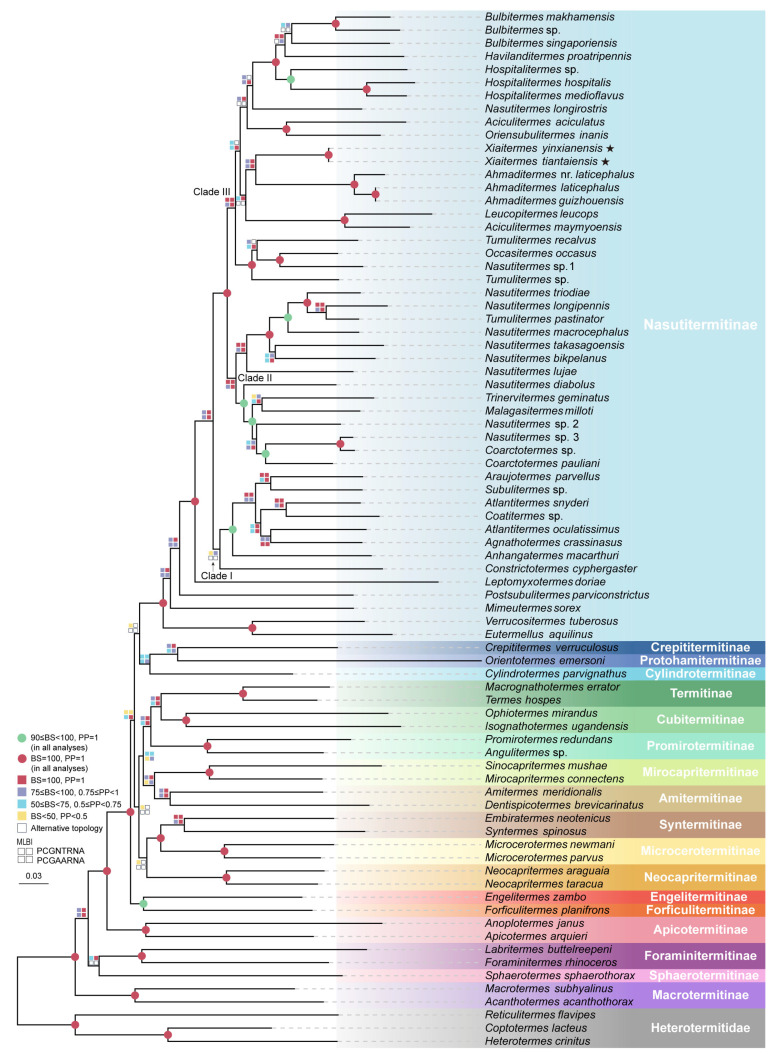
Maximum-likelihood (ML) tree of Termitidae based on the PCGNTRNA matrix. The nodes are labeled with support values: ML bootstrap values and Bayesian posterior probabilities (based on both matrices). Two *Xiaitermes* species are marked with stars.

The ML tree inferred from the PCGNTRNA matrix with support values integrated from all analyses is presented in Figure 2, and the topology details of the remaining three trees are shown in Figures S2–S4. Although the intersubfamily relationships varied somewhat among the four phylogenetic trees generated in this study, several core clades were consistently recovered. Macrotermitinae was resolved as the sister group to the other subfamilies in all of the analyses, which is consistent with all the previous phylogenetic results inferred from the mitogenome data [17,45,46]. However, the phylogenies based on nuclear genes or ultraconserved elements did not support this placement, instead suggesting that Macrotermitinae + Sphaerotermitinae together formed the most basal clade of Termitidae, with Foraminitermitinae forming a subbasal clade [2,47,48,49]. Sphaerotermitinae and Foraminitermitinae formed a sister group that diverged after Macrotermitinae (PCGNTRNA, Figure 2 and Figure S2) or diverged sequentially as independent lineages (PCGAARNA, Figures S3 and S4). Apart from the abovementioned three subfamilies, another relatively early-divergent Apicotermitinae was consistently inferred with strong support as the sister group to a clade comprising the remaining 14 subfamilies, aligning with the results from most mitogenomic or nuclear genomic datasets [2,17,46,47,48,49]. The relationships among the 14 subfamilies within the latter clade varied across the four topologies, yet they consistently shared four stable clades (Forficulitermitinae + Engelitermitinae, Syntermitinae + Microcerotermitinae, Crepititermitinae + Protohamitermitinae + Cylindrotermitinae, and Termitinae + Cubitermitinae + Promirotermitinae + Mirocapritermitinae+ Amitermitinae); the relationships among these and with Nasutitermitinae and Neocapritermitinae were discordant and poorly supported. In fact, the close phylogenetic affinities within the latter three clades were also strongly supported by genomic data [2]. Furthermore, in addition to the basal placement of Syntermitinae + Microcerotermitinae within the 14 subfamilies, the relationships among the remaining lineages remained conflicting and were not fully resolved in these phylogenomic inferences. The poorly resolved intersubfamily relationships may be attributed to rapid radiation, which allowed insufficient time for informative substitutions to accumulate, resulting in extremely short internodes among subfamilies. With the influences of partitioning schemes [46], sequence saturation and divergence heterogeneity (Figure S5), and methods of phylogenetic analyses excluded, the discrepancies in basal branches and the placement of Syntermitinae + Microcerotermitinae reflect a marked phylogenetic incongruence between mitochondrial and nuclear data, shaped by their complex evolutionary histories involving incomplete lineage sorting, introgressive hybridization or other factors [50,51].

The monophyly of Nasutitermitinae was strongly supported in all analyses, and its internal phylogenetic relationships were largely identical across the four trees. *Eutermellus aquilinus* + *Verrucositermes tuberosus* formed the basal clade as a sister group to the remaining Nasutitermitinae species, followed by the divergence of three independent lineages represented by three other African genera, *Mimeutermes*, *Postsubulitermes*, and *Leptomyxotermes*, which further supports the previous phylogenies based on limited sampling and corroborates the hypothesis of an Afrotropical origin for Nasutitermitinae [17,45]. In addition to these basal lineages, the remaining species were stably divided into a Neotropical lineage (clade I) and two other clades comprising species with multiple geographical distributions. *Xiaitermes* and *Ahmaditermes* formed a sister group with relatively strong support, which either clustered with *Leucopitermes* + *Aciculitermes* (PCRNTRNA, Figure 2 and Figure S2) or formed a sister group to other Oriental species (PCGAARNA, Figures S3 and S4) within Clade III. Although our phylogenetic analyses provided preliminary insights into the placement of *Xiaitermes*, only *Nasutitermes* was sampled among its four morphologically similar congeners. Relationships with *Malaysiotermes*, *Cucurbitermes*, and *Sinonasutitermes* could not be evaluated because of the lack of molecular data. Moreover, *Nasutitermes* has been widely shown to be non-monophyletic [15,52]. Therefore, clarifying the precise phylogenetic positions of these genera requires expanded taxon sampling, especially of Palearctic and Oriental genera/species, for further investigation.

### 3.3. Delimiting Xiaitermes spp.: Morphological Variation and Molecular Divergence

**Figure 3:**
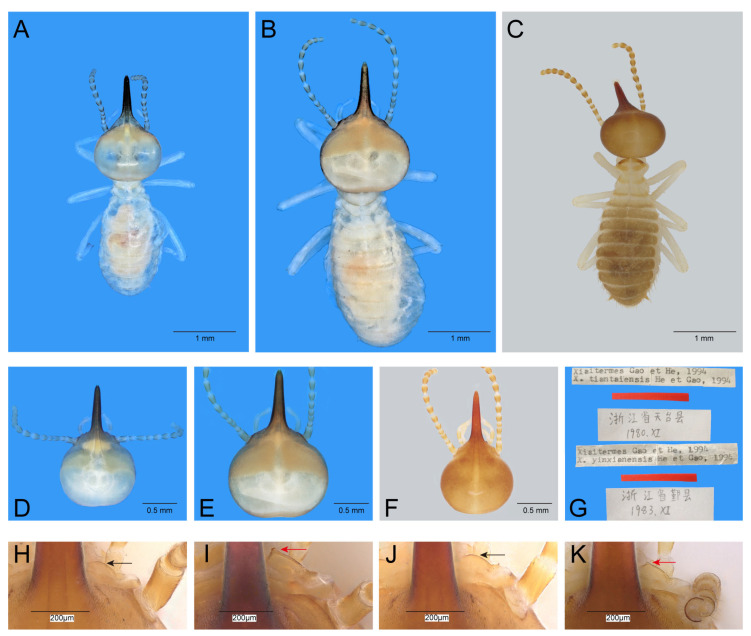
Dorsal view of *Xiaitermes tiantaiensis* and *Xiaitermes yinxianensis* soldiers. (**A**) Soldier of *X. tiantaiensis* (type specimen). (**B**) Major soldier of *X. yinxianensis* (type specimen). (**C**) Minor soldier of *X. yinxianensis*. (**D**) Soldier head of *X. tiantaiensis* (type specimen). (**E**) Major soldier head of *X. yinxianensis* (type specimen). (**F**) Minor soldier head of *X. yinxianensis*. (**G**) labels. Zhejiang Province, Tiantai County [浙江省天台县]; Zhejiang Province, Yin County [浙江省鄞县]. (**H**) Soldier mandibles of *X. tiantaiensis*. (**I**) Major soldier mandibles of *X. yinxianensis*. (**J**,**K**) Minor soldier mandibles of *X. yinxianensis*. Red and black arrows indicate the obtuse-angled and indistinct apical processes, respectively.

Morphological comparison revealed that the soldiers of *X. tiantaiensis* and the minor soldiers of *X. yinxianensis* were similar (Figure 3). Their heads are approximately broadly rounded in dorsal view, surface nearly glabrous, with only a few setae at the tip of the rostrum. Rostrums tube-like, with a small denticle-like protuberance on each side of the base. Antennae are 13-segmented, the third longer than the second and fourth. The subtle difference between the soldiers was confined to the morphology of the mandibles. The apical processes are indistinct or nearly absent in *X. tiantaiensis*. In contrast, the minor soldiers of *X. yinxianensis* exhibit morphological variation in the apical processes of mandibles among individuals from the same colony: approximately 60% of individuals possess an obtuse-angled apical process on at least one side of the mandible, whereas others display indistinct apical processes consistent with those of *X. tiantaiensis*. Compared with minor soldiers, major soldiers of *X. yinxianensis* have a broader and slightly flatter head. Antennae are 14-segmented, the second and fourth shorter than the third. The apical processes of mandibles are distinct in most individuals. In addition to the above qualitative traits, the morphological measurements (Table 1) also revealed similarities between the soldiers of *X. tiantaiensis* and the minor soldiers of *X. yinxianensis*. The ranges of the seven main morphological measurements overlapped extensively, with no significant differences.

Alignment of *COII* gene of *X. tiantaiensis* and *X. yinxianensis* revealed that they share an identical haplotype. Furthermore, the mitogenomes of both species also exhibited a high degree of similarity, with 99.9% sequence identity. A total of only 23 variable sites were identified, with the majority distributed within PCGs (Figure 1A). Among these, the *CYTB* gene contained the most variable sites, totaling five. In contrast, another widely used barcoding marker for termites, *rrnL*, contained only one variable site, which is far below the threshold for interspecific divergence [15].

**Figure 4:**
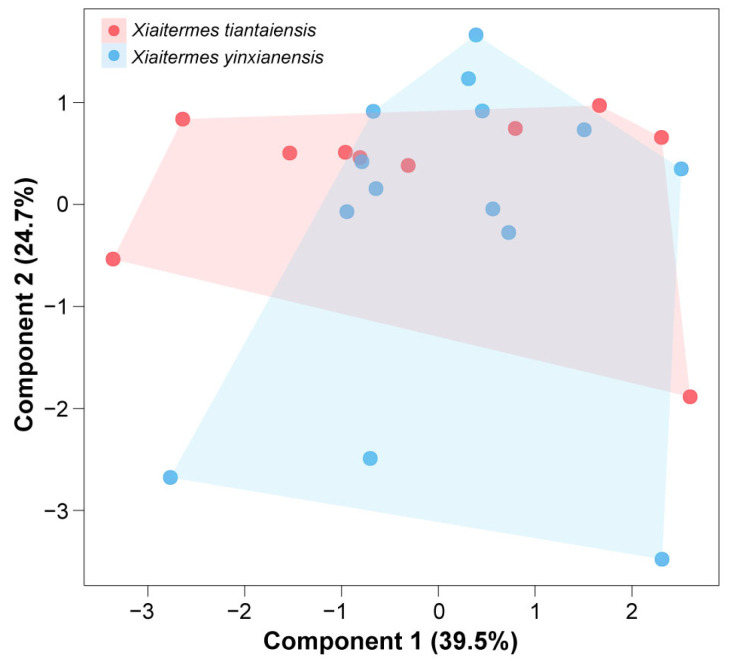
Principal component analysis of seven morphometric measurements of *Xiaitermes tiantaiensis* (soldier) and *Xiaitermes yinxianensis* (minor soldier).

On the basis of the original description [10], *X. yinxianensis* and *X. tiantaiensis* can be differentiated by the distinctness of the mandibular apical processes and body size. Although the mandibles of soldiers are generally reduced along with the highly specialized nasus within Nasutitermitinae, mandibular morphology is still used for the classification of some groups [53]. However, our morphological examination clearly revealed that the mandibular apical processes of soldiers exhibit obvious variation among individuals from the same colony and are therefore unsuitable as a primary diagnostic characteristic in this genus, which is a pattern also observed in other groups of Nasutitermitinae [13]. Furthermore, both PCA and statistical tests of the seven morphometric characteristics revealed that no soldier measurements could be used to distinguish the two species (Figure 4, Table 1). Consequently, the diagnostic characteristics, mandibular apical processes and quantitative characteristics of soldiers proposed in the original description are untenable. Combined morphological assessment and highly congruent molecular data confirmed that *X. tiantaiensis* is a junior synonym of *X. yinxianensis*.

## Figures and Tables

**Table 1:** Measurements of *Xiaitermes tiantaiensis* and *Xiaitermes yinxianensis* soldiers.

Measurement (mm)	*X. tiantaiensis* (*n* = 10)		*X. yinxianensis* (Minor Soldier, *n* = 14)		*X. yinxianensis* (Major Soldier, *n* = 13)	
	Range	Mean ± SD	Range	Mean ± SD	Range	Mean ± SD
Head length to base of mandibles	0.98–1.13	1.08 ± 0.04 A	0.95–1.10	1.05 ± 0.04 A	1.13–1.34	1.26 ± 0.06 B
Head length with rostrum	1.80–1.94	1.86 ± 0.04 A	1.81–1.92	1.84 ± 0.04 A	2.02–2.23	2.11 ± 0.07 B
Maximum head width	1.05–1.15	1.10 ± 0.03 A	1.07–1.20	1.11 ± 0.04 A	1.18–1.49	1.35 ± 0.08 B
Head height with postmentum	0.86–0.92	0.90 ± 0.02 A	0.85–0.94	0.89 ± 0.02 A	0.94–1.23	1.07 ± 0.07 B
Maximum pronotum length	0.21–0.24	0.23 ± 0.01 A	0.21–0.24	0.23 ± 0.01 A	0.26–0.37	0.31 ± 0.02 B
Maximum pronotum width	0.50–0.60	0.54 ± 0.04 A	0.50–0.61	0.53 ± 0.04 A	0.58–0.79	0.67 ± 0.05 B
Hind tibia length	1.23–1.39	1.30 ± 0.05 A	1.20–1.36	1.30 ± 0.06 A	1.31–1.73	1.57 ± 0.11 B

Note: For each character, measurements followed by the same capital letter within a row are not significantly different.

## Data Availability

The mitochondrial genomes generated in this study are available from NCBI under accession numbers PZ210214 and PZ210215.

## Supplementary Materials

The following supporting information can be downloaded at https://www.mdpi.com/article/10.3390/insects17060602/s1: Figure S1: Secondary structure of *Xiaitermes tiantaiensis* and *Xiaitermes yinxianensis* mitochondrial tRNAs. Figure S2: Bayesian tree of Termitidae based on the PCGNTRNA matrix. The posterior probabilities below 0.5 are not shown. Figure S3: Maximum-likelihood tree of Termitidae based on the PCGAARNA matrix. The bootstrap values below 50 are not shown. Figure S4: Bayesian tree of Termitidae based on the PCGAARNA matrix. Figure S5: AliGROOVE analysis for protein-coding genes (PCGs), rRNA genes, and tRNA genes. Amino acid sequences of PCGs (A). Nucleotide sequences of PCGs (B), rRNA genes (C) and tRNA genes (D). Table S1: List of samples used in phylogenetic analyses. References [54,55,56,57,58,59,60] are cited in the Supplementary Materials.
