# Supplementary figures and images for: Revisiting a Chinese Endemic Termite Genus of Building Timber Pests: Mitochondrial Genomic and Morphological Dissection and Phylogenetic Positioning of Xiaitermes Gao & He, 1994 (Termitidae: Nasutitermitinae)
Source: Insects. 2026 Jun 8;17(6):602. doi: 10.3390/insects17060602 (PMC13300816; doi:10.3390/insects17060602)

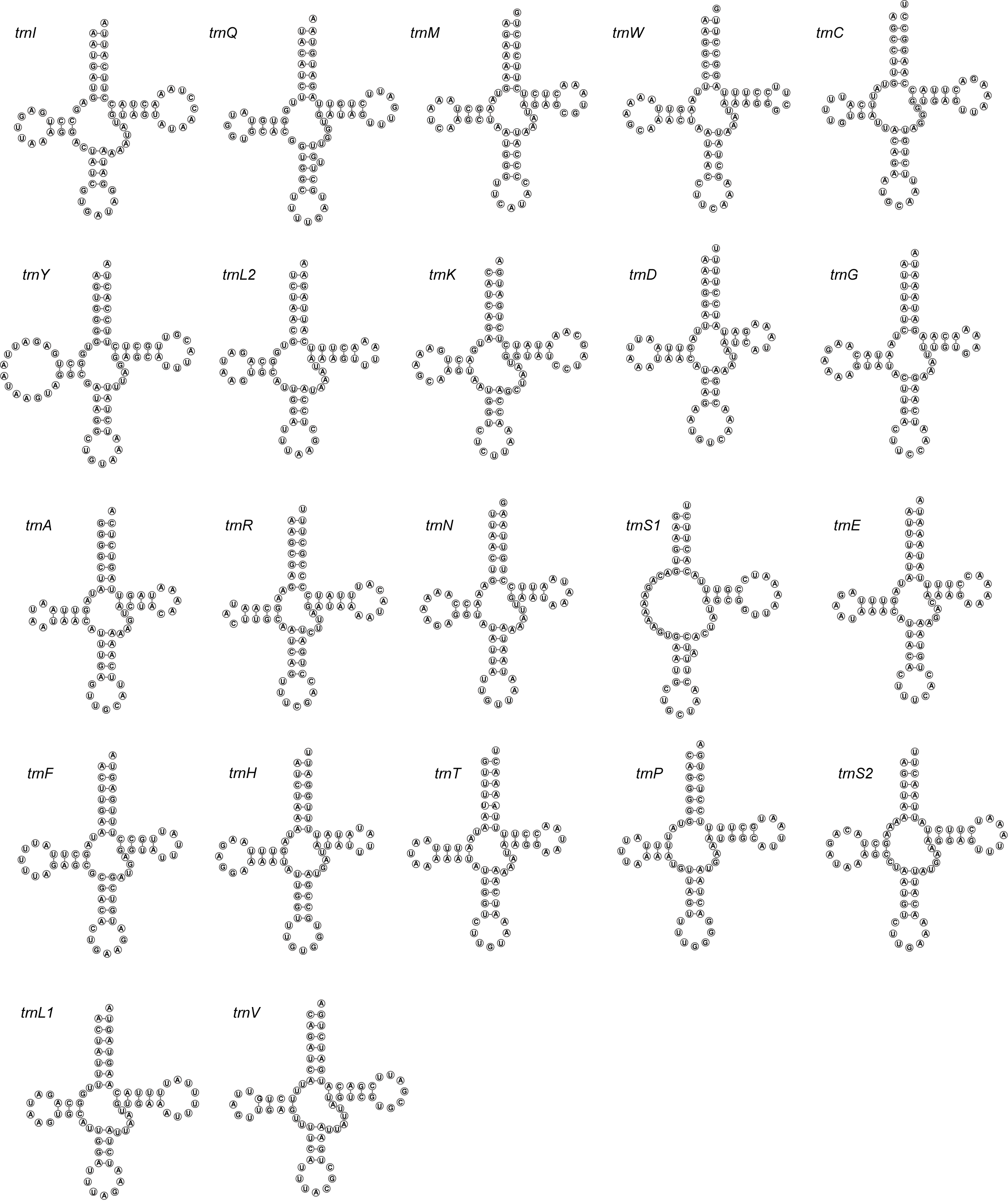

Supplement: Supplementary file 1 [file insects-17-00602-s001.zip › Figure S1.tif]

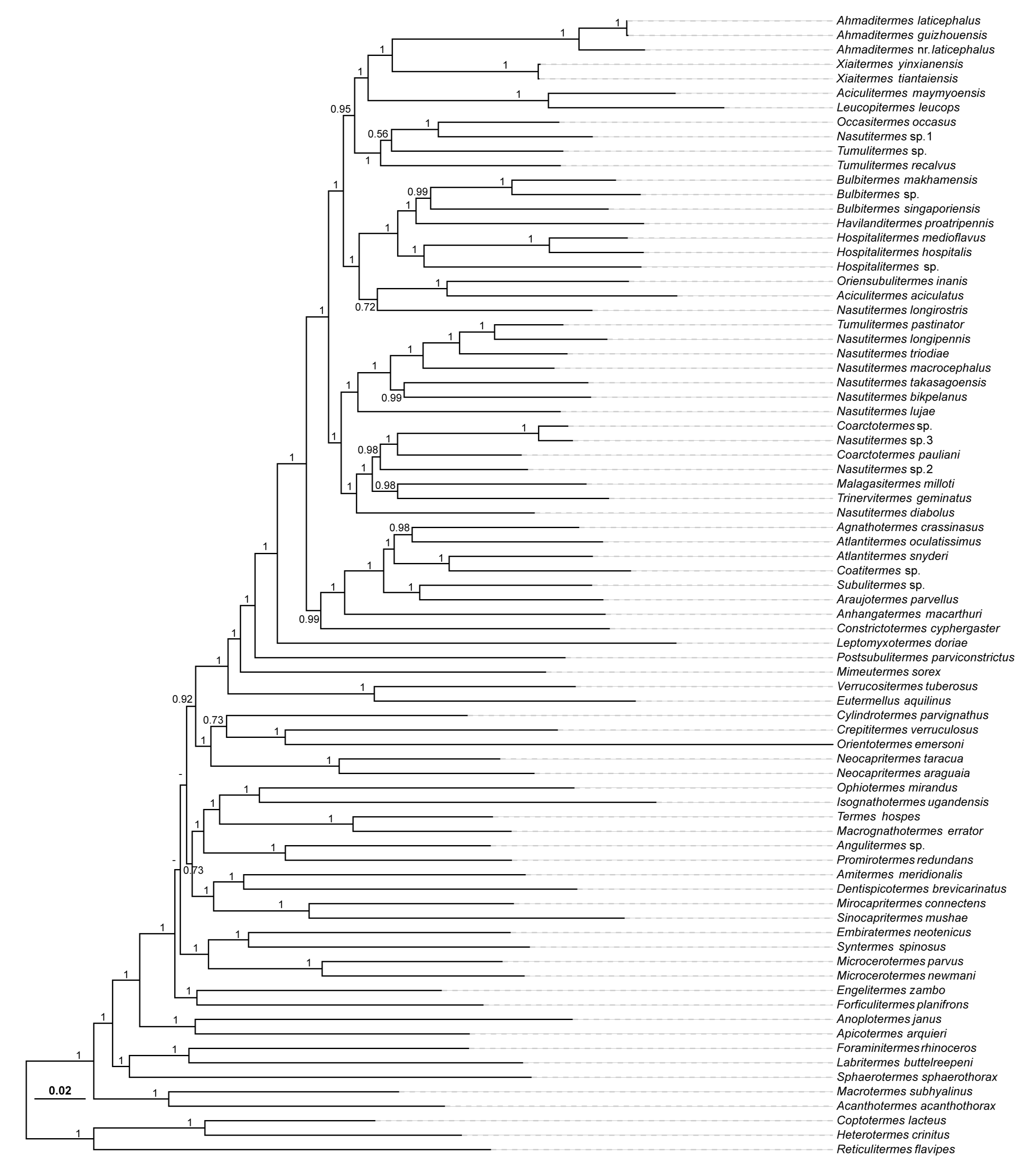

Supplement: Supplementary file 1 [file insects-17-00602-s001.zip › Figure S2.tif]

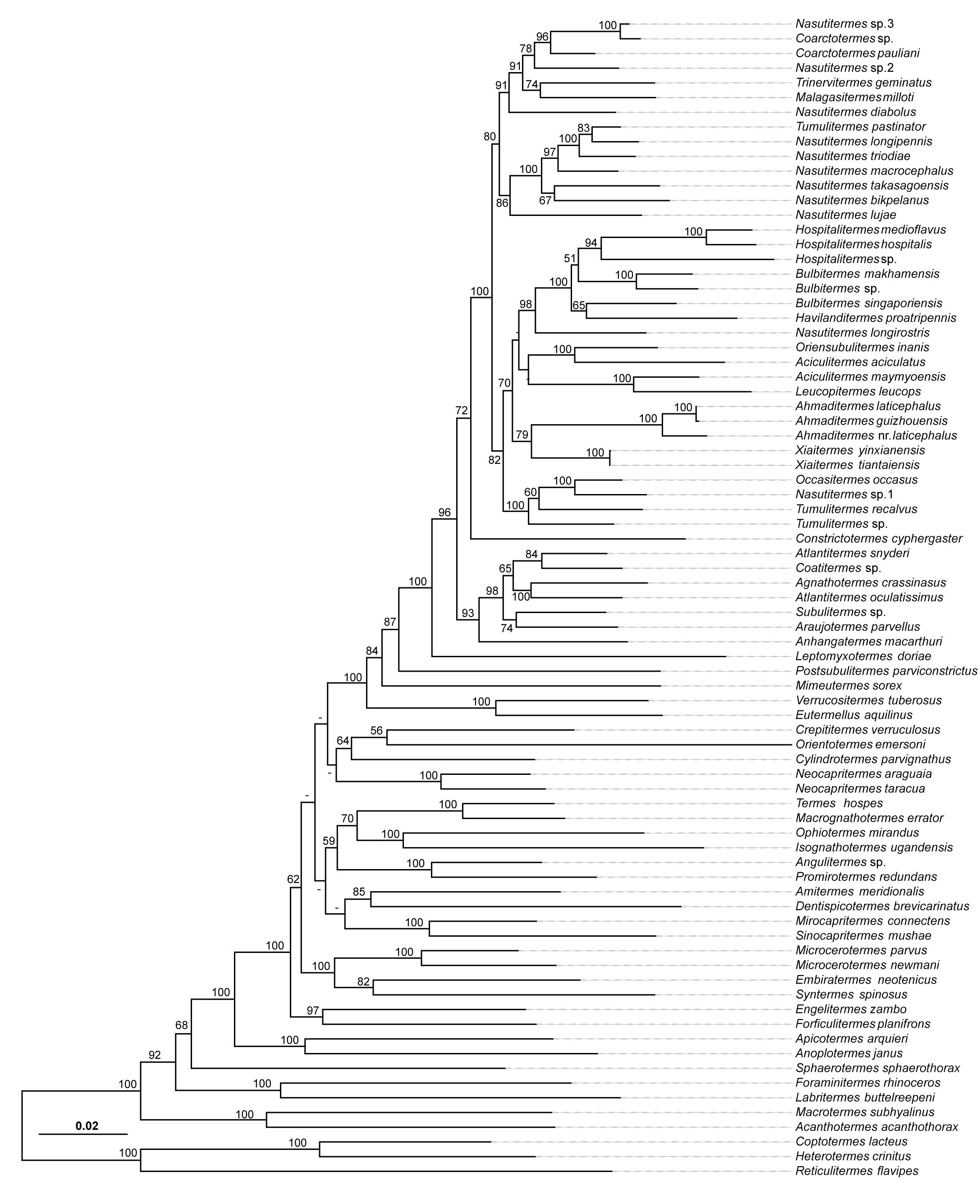

Supplement: Supplementary file 1 [file insects-17-00602-s001.zip › Figure S3.tif]

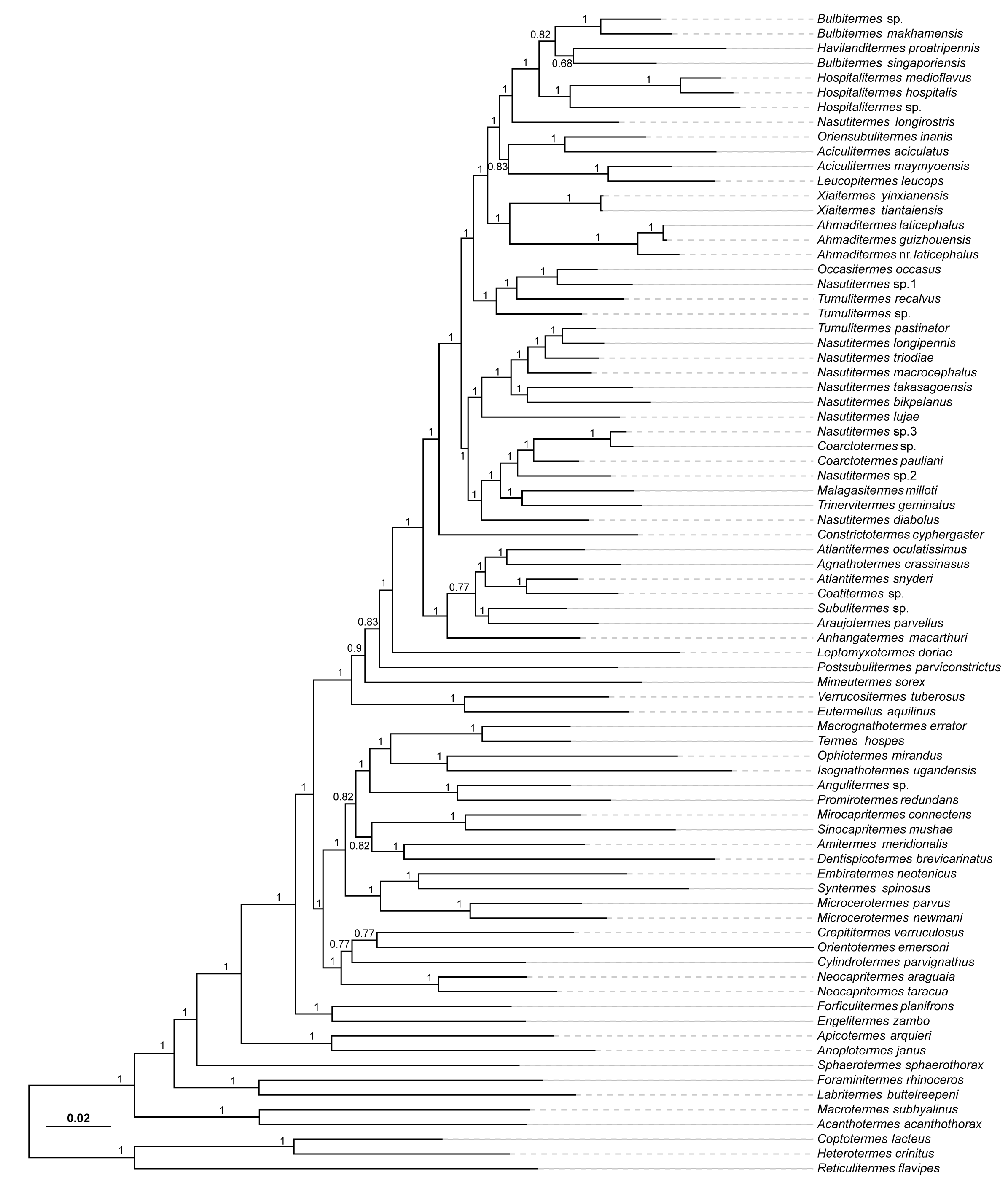

Supplement: Supplementary file 1 [file insects-17-00602-s001.zip › Figure S4.tif]

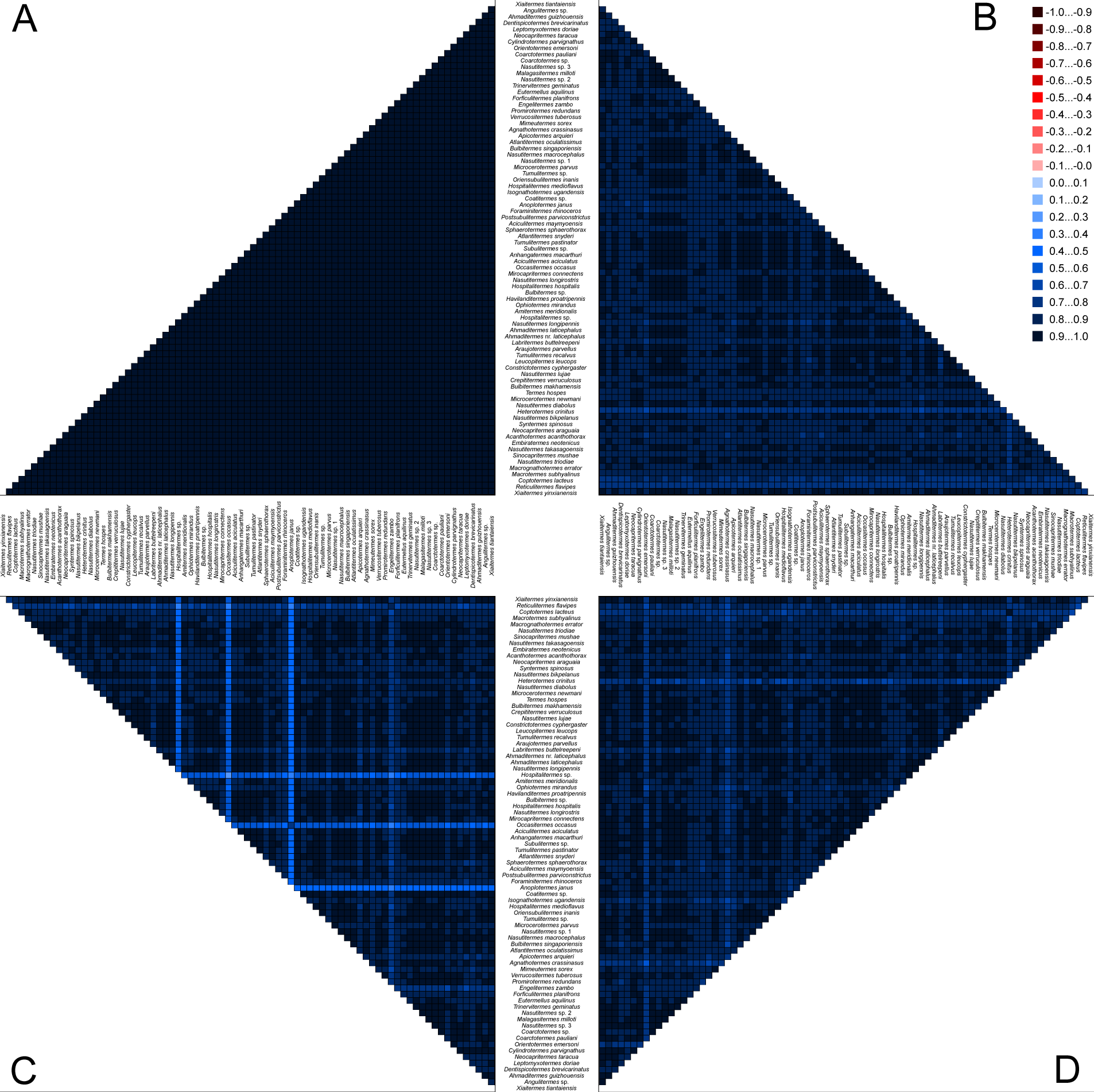

Supplement: Supplementary file 1 [file insects-17-00602-s001.zip › Figure S5.tif]
